# Mechanism of Pterostilbene-Induced Cell Death in HT-29 Colon Cancer Cells

**DOI:** 10.3390/molecules27020369

**Published:** 2022-01-07

**Authors:** Joanna Wawszczyk, Katarzyna Jesse, Sławomir Smolik, Małgorzata Kapral

**Affiliations:** 1 Department of Biochemistry, Faculty of Pharmaceutical Sciences in Sosnowiec, Medical University of Silesia, Jedności 8, 41-200 Katowice, Poland; ssmolik@sum.edu.pl; 2 Prof. Z. Religa Foundation of Cardiac Surgery Development, Heart Prostheses Institute, Wolności 345a, 41-800 Zabrze, Poland; kjesse@frk.pl; 3 Silesian Park of Medical Technology Kardio-Med Silesia, M. Curie-Skłodowskiej 10C, 41-800 Zabrze, Poland

**Keywords:** pterostilbene apoptosis, autophagy, AKT, STAT3, colon cancer

## Abstract

Pterostilbene is a dietary phytochemical that has been found to possess several biological activities, such as antioxidant and anti-inflammatory. Recent studies have shown that it exhibits the hallmark characteristics of an anticancer agent. The aim of the study was to investigate the anticancer activity of pterostilbene against HT-29 human colon cancer cells, focusing on its influence on cell growth, differentiation, and the ability of this stilbene to induce cell death. To clarify the mechanism of pterostilbene activity against colon cancer cells, changes in the expression of several genes and proteins that are directly related to cell proliferation, signal transduction pathways, apoptosis, and autophagy were also evaluated. Cell growth and proliferation of cells exposed to pterostilbene (5–100 µM) were determined by SRB and BRDU assays. Flow cytometric analyses were used for cell cycle progression. Further molecular investigations were performed using quantitative real-time RT-PCR. The expression of the signaling proteins studied was determined by the ELISA method. The results revealed that pterostilbene inhibited proliferation and induced the death of HT-29 colon cancer cells. Pterostilbene, depending on concentration, caused inhibition of proliferation, G1 cell arrest, and/or triggered apoptosis in HT-29 cells. These effects were mediated by the down-regulation of the STAT3 and AKT kinase pathways. It may be concluded that pterostilbene could be considered as a potential therapeutic option in the treatment of colon cancer in the future.

## 1. Introduction

Globally, colorectal cancer (CRC) is the third most commonly diagnosed malignancy and the second leading cause of cancer-related mortality. According to Global Cancer Statistics, more than 1.8 million new CRC cases and 881,000 deaths were estimated to occur in 2018 [1]. The incidence of CRC has been the highest in wealthy Western countries, but it is now increasing rapidly with economic development in other parts of the world [2]. The most important risk factors for CRC are associated with westernization (e.g., environmental factors, diet, sedentary lifestyle, alcohol). Furthermore, the increasing incidence of CRC at younger ages (before the age of 50 years) is an emerging trend [3]. CRC is an etiologically heterogeneous disease that is subtyped by the tumor anatomical location or global molecular alterations. There are known three major pathogenetic pathways of this cancer, including adenoma–carcinoma sequence, serrated pathway, and inflammatory pathway [3]. Therefore, it is important to discover inexpensive natural compounds to be effective in preventing and suppressing the destructive effects of colon tumors.

Recently, traditional medicines, which are derived from natural compounds, have been reported as promising therapeutic agents. In particular, polyphenols such as resveratrol possess attractive features for use in cancer prevention and treatment. Among natural polyphenols, stilbenoids, which occur in blueberries and grapes, represent a class of non-flavonoid polyphenolic compounds that have been extensively studied in recent years [4]. They are known to possess a wide range of biological activities, including antioxidant, anti-inflammatory, antifungal, and antibacterial effects in vitro and in vivo through pleotropic mechanisms [5,6]. Pterostilbene (trans-3,5-dimethoxy-4′hydroxystilbene) is a natural analog of resveratrol, showing a higher bioavailability than the latter [7]. The results of some studies suggest that it may be a promising chemotherapeutic agent [6,8]. The complex chemistry of pterostilbene allows it to inhibit multiple oncogenic processes [6]. Pterostilbene has been shown to protect cells from oxidative damages [9]. Other studies have revealed its cytotoxic and growth inhibitory effect on several types of cancer cells such as liver [10], lung [11], or breast [12] cells. Moreover, pterostilbene-induced cell death may occur via different mechanisms [13]. These biological effects lay the foundation for their potential application in the treatment of cancers as well.

Therefore, the main goal of the study was to investigate the anticancer activity of pterostilbene against human colon cancer cells, focusing on its influence on their proliferation, differentiation, and death. Furthermore, the study aimed at assessing whether pterostilbene induces apoptosis and autophagy of colon cancer cells, and finally, to understand the role of some molecular pathways in the biological activity of this compound.

## 2. Results

### 2.1. Growth Inhibition of HT-29 Pterostilbene

**Figure 1:**
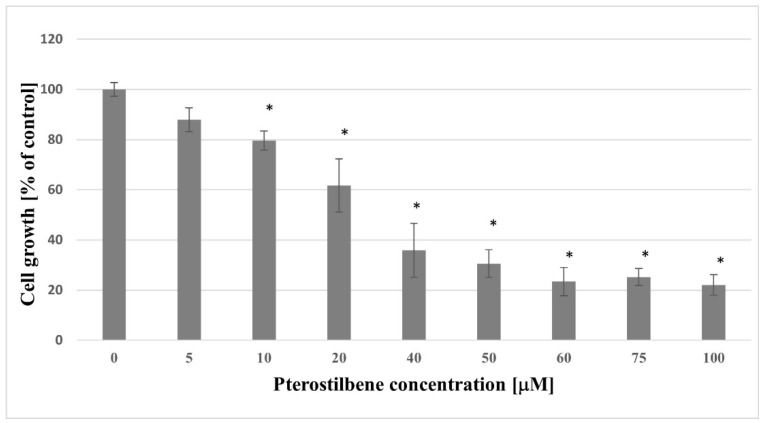
Growth inhibitory effect of pterostilbene on HT-29 cells after 48 h of treatment. The results are expressed as a percentage of untreated control (the means ± SD; * *p* < 0.05 vs. control).

In the present study, HT-29 cells were used as the colon cancer cell model in vitro. The design of the experiments focused firstly on investigating the response of pterostilbene treated colon cancer cells. Additionally, the effect of pterostilbene on cellular growth was evaluated using the SRB assay. The effect of DMSO on the growth of the HT-29 cell line was also evaluated by the culture of cells in a medium containing 0.1% DMSO, but no influence of this compound was found (data not shown). Cells were exposed to increasing concentrations of pterostilbene (5–100 µM) for 48 h (Figure 1). The lowest concentration of pterostilbene (5 µM) did not affect colon cancer cell growth. The statistically significant decrease in HT-29 cell growth was evoked by pterostilbene at higher concentrations (≥10 µM). The most pronounced reduction in cellular density was observed in cell cultures treated with 100 μM of pterostilbene. Taken together, these data demonstrated that pterostilbene decreased the growth of HT-29 cells in a dose-dependent manner.

### 2.2. Antiproliferative Activity of Pterostilbene

**Figure 2:**
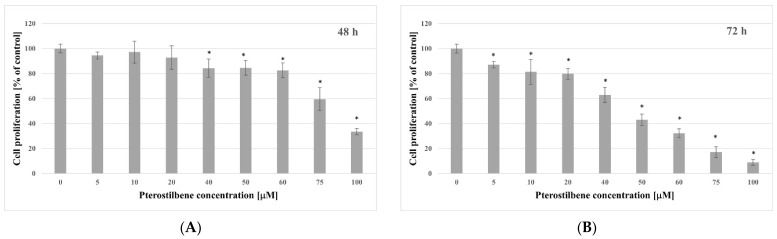
Antiproliferative effect of pterostilbene on HT-29 cells after (**A**) 48 h and (**B**) 72 h treatment. The results are expressed as a percentage of untreated control (the means ± SD; * *p* < 0.05 vs. control).

The BrdU incorporation assay was used to determine newly synthesized DNA to indicate the effect of pterostilbene on HT-29 cell proliferation after 48 and 72 h. The results showed that there was a significant decrease in BrdU incorporation in a dose-dependent manner after incubation with pterostilbene (Figure 2). The effect of this compound on the proliferation of HT-29 cells after 48 h is shown in Figure 2A. Its concentrations ≤ 20 µM did not affect HT-29 cells proliferative activity, and higher concentrations (40–100 µM) significantly decreased proliferation. The inhibitory effect of pterostilbene on HT-29 cell proliferation was increased by time exposure, particularly at low concentrations. When the experimental time period was extended to 72 h, pterostilbene at all concentrations used (5–100 µM) caused significant inhibition of proliferation, and the maximum growth reduction (91%) was observed at 100 µM (Figure 2B). Taken together, these data demonstrated that pterostilbene decreased the proliferation rate of HT-29 cells in a dose- and time-dependent manner.

### 2.3. The Effect of Pterostilbene on Differentiation

**Figure 3:**
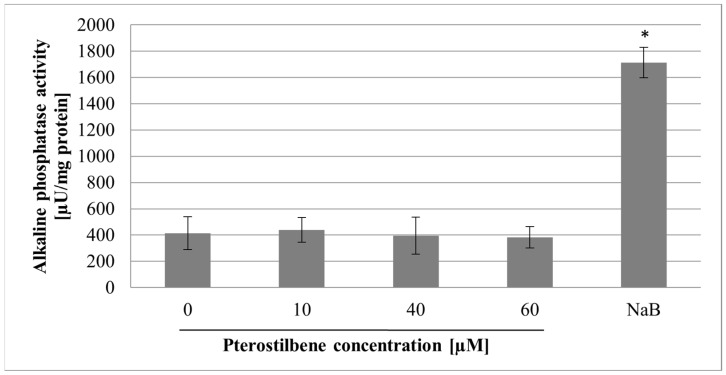
Effect of pterostilbene (10–60 µM) and sodium butyrate (NaB) (1 mM) on alkaline phosphatase (ALP) activity after 72 h of treatment. Results are presented as mean ± SD; * *p* < 0.05 vs. control.

A cell division arrest is one of the prerequisites for cell differentiation. In the study, the effect of pterostilbene on HT-29 cells differentiation was determined by measuring alkaline phosphatase (ALP) activity, a marker of the degree of colonocyte differentiation [14]. Sodium butyrate (NaB), a natural product of the colonic bacterial flora, has been reported to increase the activity of ALP in colon cancer cells, so it was used as a positive control. The effect of the compound studied on ALP activity is shown in Figure 3. As expected, NaB (at concentration 1 mM) caused a significant, over 4-fold increase of ALP activity as compared with control. However, pterostilbene at all concentrations used (10, 40, and 60 µM) did not affect the ALP enzyme activity (*p* > 0.05). 

### 2.4. The Effect of Pterostilbene on HT-29 Cell Cycle Distribution

**Figure 4:**
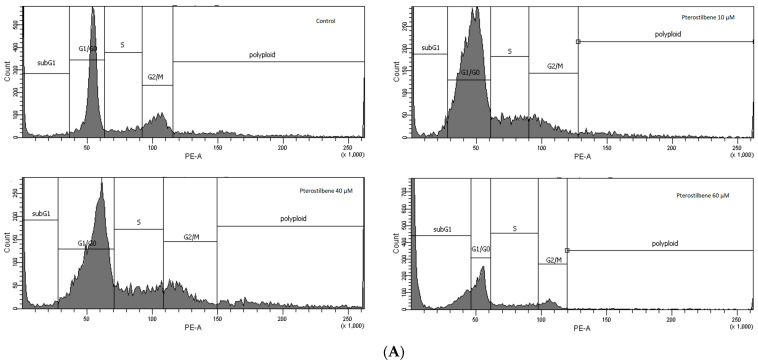

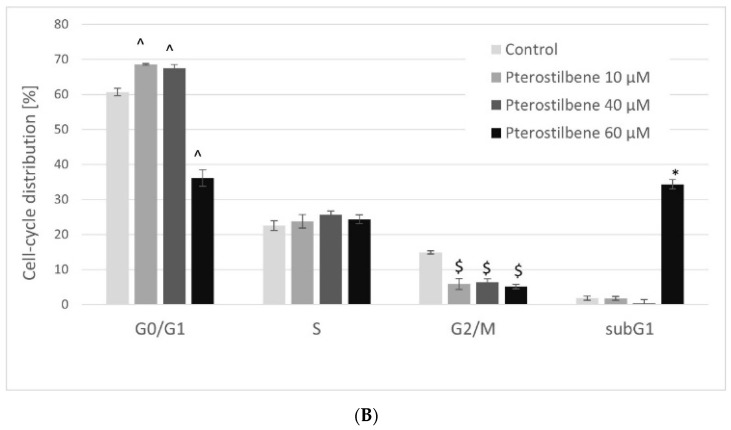
The effect of pterostilbene on HT-29 cell cycle after 72 h. The cells were labeled with PI for DNA contents and analyzed by flow cytometry (**A**) Representative histograms of cell cycle analysis (**B**) Cell cycle distribution. The data indicate the percentage of cells in each phase of cell cycle. (the means ± SD; ˆ (G0/G1), $ (G2/M), * (subG1), *p* < 0.05 vs. control).

To recognize whether the reduced cell viability was due to inhibition of cell cycle progression, flow cytometry was used. The effect of pterostilbene on the cell cycle was investigated after 72 h. As shown in Figure 4, pterostilbene at all concentrations used reduced the number of HT-29 cells in the G2/M phase. Exposure of cells to pterostilbene at lower concentrations (10 and 40 µM) increased the percentage of cells in the G0/G1 phase. Treatment of cells with 60 μM pterostilbene resulted in a significant increase in the population of cells in the sub-G1 phase, which further suggested that it induced cancer cell apoptosis. These results suggested that the mechanism of pterostilbene activity may depend on its concentration. It might arrest colon cancer cells in the G1 phase at low concentrations and increase the sub-G1 phase at the highest concentration.

### 2.5. Transcriptional Activity of Genes Encoding the Cell Cycle Regulating Proteins in HT-29 Cells Exposed to Pterostilbene

Quantitative RT-PCR to evaluate the effect of pterostilbene on the proliferation of colon cancer cells was performed. HT-29 cells were incubated with pterostilbene for 6 and 12 h. Next, the transcription level of the *CCND1* and *CDKN1A* genes encoding key proteins of cell cycle CD1 and p21Waf1/Cip1, respectively, was examined. 

**Figure 5:**
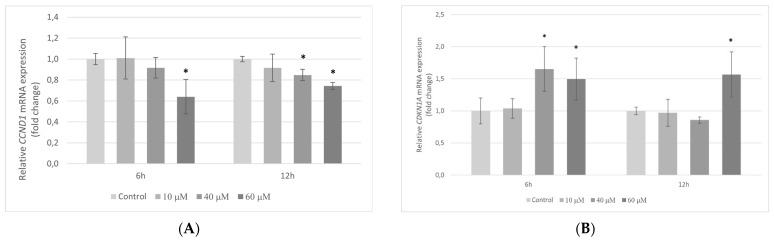
Expression of (**A**) *CCND1*; (**B**) *CDKN1A* genes in HT-29 cells after treatment with 10, 40, and 60 µM pterostilbene for 6 h and 12 h. The results are presented as mean ± SD; * *p* < 0.05 vs. control.

Pterostilbene at lower concentrations (10 and 40 µM) had no influence on *CCND1* expression at 6 h. On the contrary, treatment of cells with 60 µM pterostilbene significantly decreased the amount of *CCND1* mRNA in comparison to control. After 12 h, a marked decrease in *CCND1* gene transcription was observed in cultures treated with pterostilbene at both 40 and 60 µM and pterostilbene at concentration 60 µM evoked a stronger effect (Figure 5A).

At 6 h, 10 µM pterostilbene had no effect on *CDKN1A* transcription; however, higher concentrations of this compound significantly up-regulated gene expression in relation to control. At a longer period (12 h), a significant increase in the level of *CDKN1A* mRNA was observed with 60 µM pterostilbene only (Figure 5B). 

### 2.6. The Impact of Pterostilbene on The p21 Protein Level 

**Figure 6:**
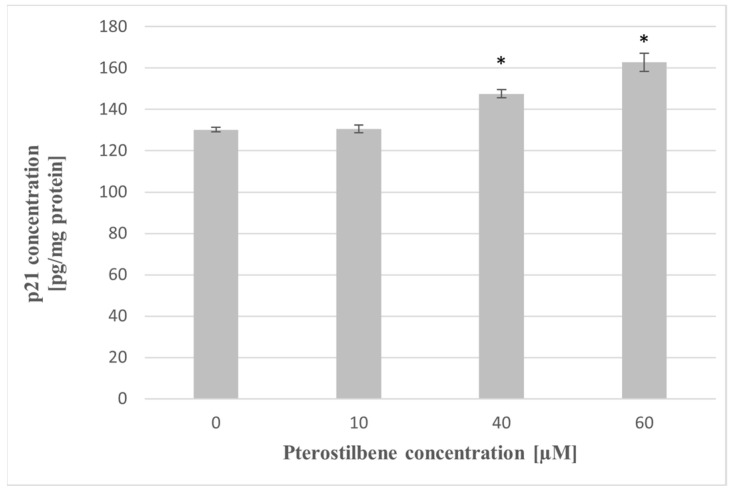
Effect of pterostilbene on p21 concentration in HT-29 cells after 24 h of incubation. The results are presented as mean ± SD; * *p* < 0.05 vs. control.

To determine whether the induction of *CDKN1A* mRNA expression corresponds with the p21 protein level at the next step of the study, the effect of pterostilbene on the concentration of p21 protein in HT-29 cells has been evaluated. Exposure of cells to pterostilbene at concentrations 40 and 60 µM for 24 h resulted in up-expression of the p21 protein compared to untreated cells (Figure 6). These findings suggest that the mechanism of pterostilbene activity on HT-29 cells is elucidated by increased expression of the *p21* gene, a cyclin kinase inhibitor, at both the mRNA and protein levels.

### 2.7. The Influence of Pterostilbene on mRNA Expression of Genes Encoding AKT and STAT3 Proteins in HT-29 Cells

**Figure 7:**
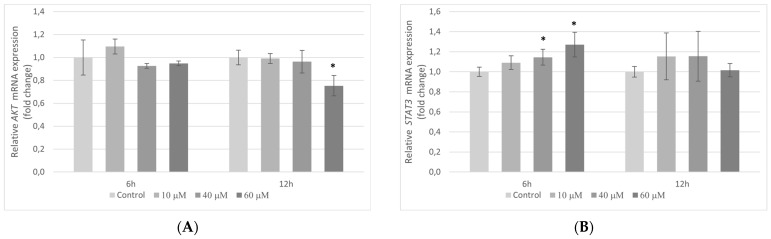
Effect of pterostilbene on the expression of (**A**) *AKT* and (**B**) *STAT3* genes in HT-29 cells after treatment with 10, 40, and 60 µM pterostilbene for 6 h and 12 h. The results are presented as mean ± SD; * *p* < 0.05 vs. control.

Taking into account the widely recognized role of *AKT* and *STAT3* signaling in cancer cell proliferation, a possible modulation of this signaling in HT-29 cells by pterostilbene was also evaluated. As can be seen in Figure 7A, cells treated with pterostilbene for 6 h did not manifest any changes in *AKT* mRNA level. However, longer incubation with 60 µM pterostilbene (12 h) markedly decreased *AKT* transcription in HT-29 cells. By comparison, at 6 h pterostilbene up-regulated transcriptional activity of *STAT3* gene in colon cancer cells in relation to control, whereas no marked changes in *STAT3* gene activity were demonstrated in cells treated with pterostilbene for 12 h (Figure 7B).

### 2.8. The Effect of Pterostilbene on AKT Activity 

**Figure 8:**
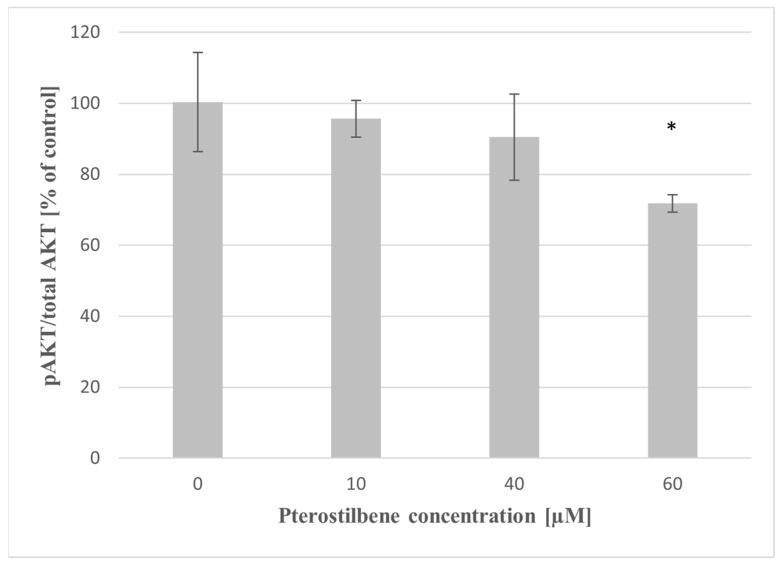
The influence of pterostilbene on AKT activity in HT-29 at 24 h. The results are presented as mean ± SD; * *p* < 0.05 vs. control.

To further examine whether the mechanism of action of pterostilbene in colon cancer cells involves the regulation of AKT kinase, we analyzed the effect of pterostilbene on AKT phosphorylation in HT-29 cells. As shown in Figure 8, pterostilbene at 60 µM concentration markedly inhibited p-AKT/total AKT ratio after 24 h compared to untreated cells. Lower concentrations of pterostilbene also suppressed this phosphorylation; however, the comparative analysis did not reveal it significant. 

### 2.9. The Influence of Pterostilbene on STAT3 Activity 

**Figure 9:**
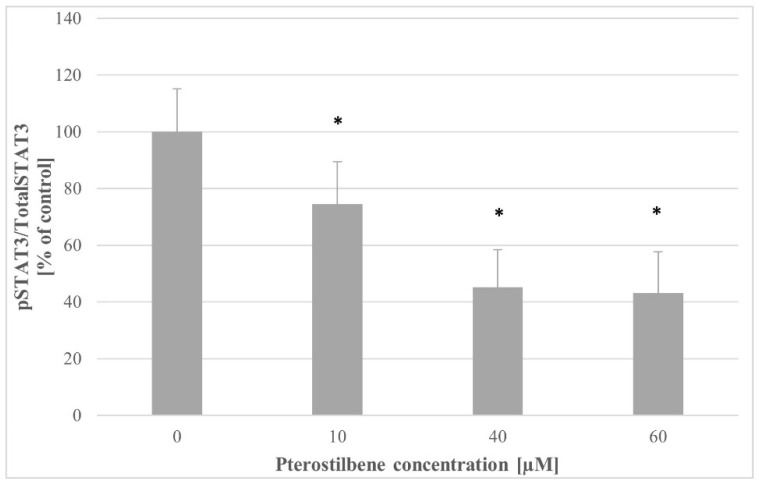
Effect of pterostilbene on STAT3 activity in HT-29 cells at 24 h. The results are presented as mean ± SD; * *p* < 0.05 vs. control.

To evaluate whether the STAT3 pathway could be changed with pterostilbene treatment, the p-STAT3/total STAT3 ratio was determined in control cells and cells incubated with pterostilbene. As shown in Figure 9, pterostilbene at all concentrations used caused an essential down-regulation of STAT3 in HT-29 cells compared to the control. There were no significant differences between STAT3 activity in cultures treated with 40 and 60 µM pterostilbene.

### 2.10. The Effect of Pterostilbene on HT-29 Cells Apoptosis

**Figure 10:**
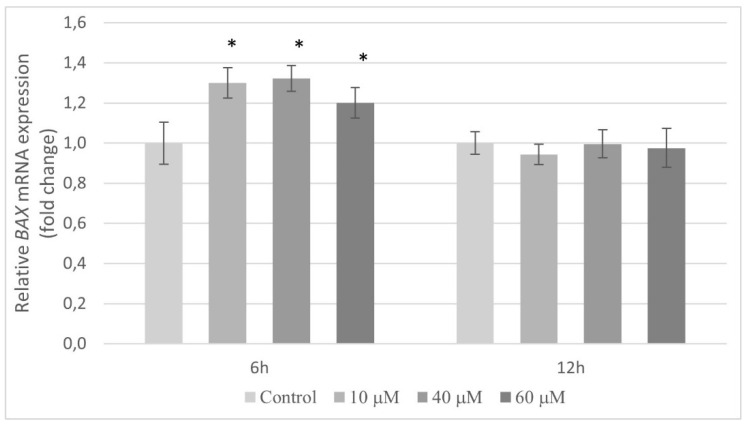
Expression of the *BAX* gene in HT-29 cells after treatment with 10, 40, and 60 µM pterostilbene for 12 and 24 h. The results are presented as mean ± SD; * *p* < 0.05 vs. control.

The expression of the *BAX* gene in relation to apoptotic pathways has been analyzed. Compared to the control, pterostilbene at all concentrations used caused markedly induction of the *BAX* mRNA in HT-29 cells after 6 h. However, at a longer period (12 h), this compound had no effect on the transcription of the gene encoding *BAX* protein (Figure 10). 

**Figure 11:**
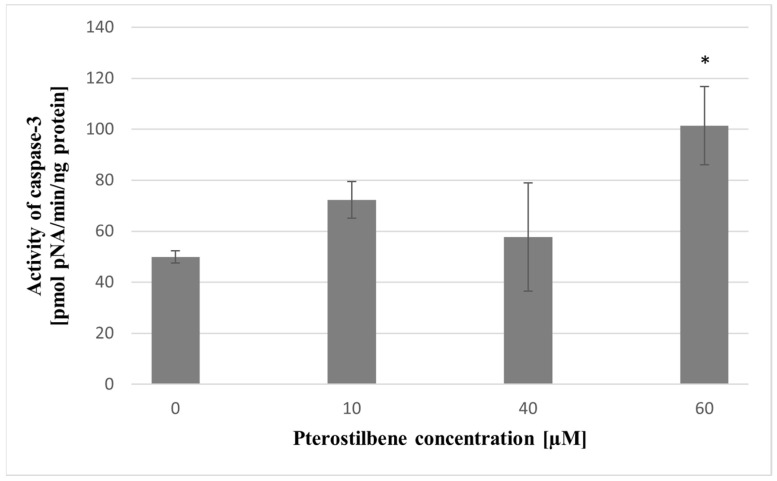
Effect of pterostilbene at concentrations of 10, 40, and 60 µM on caspase-3 activity in HT-29 cells at 48 h. The results are presented as mean ± SD; * *p* < 0.05 vs. control.

In order to confirm the early stages of apoptosis, the impact of pterostilbene on caspase-3 activity in colon cancer cells was estimated. In cells incubated with 10 and 40 µM pterostilbene, caspase activity did not alter when compared to untreated cells. Exposure of cells to the highest concentration (60 µM) resulted in a significant increase in caspase-3 activity in relation to control (Figure 11). 

**Figure 12:**
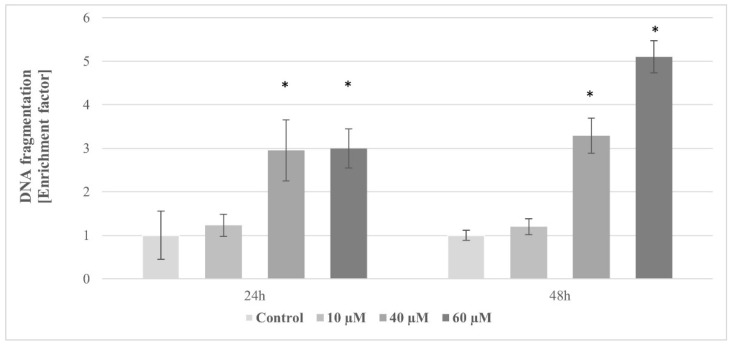
The specific enrichment of mononucleosomes and oligonucleosomes released into the cytoplasm of the cells treated with pterostilbene at concentrations of 10, 40, and 60 µM for 24 and 48 h. The results are presented as mean ± SD; * *p* < 0.05 vs. control.

To confirm the induction of apoptosis on pterostilbene treated cultures, Cell Death ELISA was performed to quantify DNA fragmentation levels, which is a major hallmark of apoptosis (Figure 12). A significant dose-dependent increase in enrichment factor was noted in HT-29 cells incubated with pterostilbene for both 24 h and 48 h. A statistically significant 3-fold increase in enrichment factor was observed in cells treated with 40 and 60 µM pterostilbene for 24 h (Figure 12). Prolonged incubation caused a further increase in the concentration of histone-associated DNA in cells treated with the highest concentration of the stilbene. 

### 2.11. The Influence of Pterostilbene on the Transcriptional Activity of Autophagy-Related Genes in HT-29 Cells

**Figure 13:**
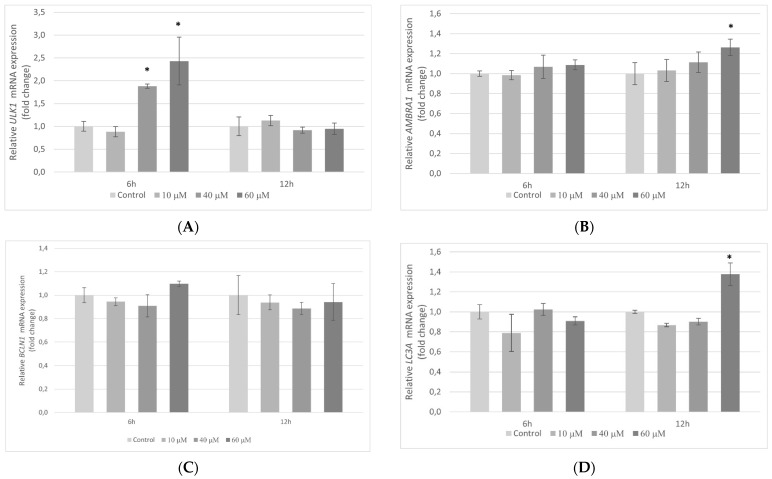
Expression of the (**A**) *ULK1*, (**B**) *AMBR1*, (**C**) *BCLN1*, and (**D**) *LC3A* genes in HT-29 cells after treatment with pterostilbene for 6 and 12 h. The results are presented as mean ± SD; * *p* < 0.05 vs. control.

To establish whether pterostilbene may influence autophagy-related genes, transcriptional activities of the *ULK1, AMBRA1, BCLN1,* and *LC3A* genes in colon cancer cells treated with pterostilbene for 6 and 12 h were examined. Pterostilbene at a lower concentration (10 µM) did not affect the expression of the genes studied in HT-29 cells. However, higher concentrations of this compound modulated the amount of *ULK1, AMBRA1*, and *LC3A* mRNAs. (Figure 13A,B,D). Pterostilbene at both 40 and 60 µM markedly increased the level of *ULK1* mRNA in comparison to control at 6 h. At a longer time period (12 h), pterostilbene at concentration 60 µM significantly induced *AMBRA1* and *LC3A* mRNAs. Pterostilbene at all concentrations used had no effect on *BCLN1* expression at both studied time periods (Figure 13C).

## 3. Discussion

Colorectal adenocarcinoma is one of the most common cancers worldwide, with high mortality [1,15]. Due to its aggressive nature and poor response to chemotherapy, colon cancer remains a challenging disease to treat. Therefore, novel, more effective treatment strategies need to be developed. A number of studies point to the influence of diet and dietary factors on the colon cancer risk [15,16]. In previous years, a plethora of phytochemicals, such as polyphenols, have been reported to possess an inhibitory effect on the development of multiple cancers [17,18]. Polyphenols are considered a viable option in the treatment of cancer due to their potent anticancer action and no or less adverse effects [19]. The anticancer activity of polyphenolic compounds is mediated through a variety of mechanisms, including influence on signaling pathways associated with cell survival, proliferation, differentiation, antioxidant enzymes, and immune responses [20]. One of the most extensively studied polyphenols is resveratrol. Notwithstanding their promising role in the prevention and treatment of several types of cancer, it has poor bioavailability. Its oral administration encounters high metabolism leading to low levels of free resveratrol in the serum [21]. 

Pterostilbene is a methoxylated analog of resveratrol. The difference in chemical structures causes pterostilbene to be more lipophilic, which enhances its membrane permeability and bioavailability [22]. The results of many studies suggest that this component of berries and grapes may be a promising chemotherapeutic agent against different cancers, including colorectal cancer [4,8]. Several in vivo studies have shown that dietary administration of pterostilbene reduced colon tumor multiplicity in rats [23] and inhibited the formation of azoxymethane (AOM)-induced aberrant crypt foci and adenomas in mice [24]. Furthermore, the expression of proliferating cell nuclear antigen (PCNA) was reduced in colon tumors from pterostilbene-fed animals [22]. Cancer progression is a consequence of uncontrolled DNA replication and cell division, avoidance of programmed cell death, invasion of local tissues, and metastasis. Cancer cell survival is the result of an imbalance between their proliferation and death [25]. It has been revealed that cell cycle regulators are frequently mutated in malignant cells; therefore, the main goal of cancer treatment is to control cancer cell cycle progression and to promote their death [25,26]. Since assessment of cancer cell proliferation and death is a key component in the discovery and development of anticancer drugs in the current study, we investigated changes in cell growth of HT-29 human colon cancer cells after incubation with pterostilbene as well as the ability of this stilbene to the induction of HT-29 cell death. To clarify the mechanism of pterostilbene activity against colon cancer cells, we also evaluated the changes in the expression of several genes and proteins that are directly related to cell proliferation, signal transduction pathways, apoptosis, and autophagy.

The present results indicate anticancer effects of pterostilbene on HT-29 cells. In detail, the cell growth assay showed that pterostilbene at concentrations ≥10 µM demonstrated growth inhibitory effect on HT-29 cells after 48 h treatment. Natakul et al. [27] also demonstrated the reduction of HT-29 cell growth after 48 h exposure to pterostilbene with the IC50 of about 15 μM. Other research has also confirmed the reduction of colon cancer cell growth exerted by pterostilbene. The time- and dose-dependent growth inhibitory effect of pterostilbene against six different colon cancer cell lines (CL187, COLO205, HCT-8, SW480, LoVo, and HCT-116) was observed in studies performed by Zhang et al. [28], and after 48 h treatment with pterostilbene, the IC50 values ranged from 13.82 and 35.73 µM. Furthermore, the results of our study revealed not only inhibition of growth but also a significant time- and dose-dependent reduction in the proliferation rate of HT-29 after incubation with pterostilbene. Other research supports the antiproliferative activity of this stilbene against human colon cancer cells. The HT-29 cells incubated with pterostilbene caused a dose-dependent reduction of [3H]thymidine incorporated into DNA [29]. Interestingly, pterostilbene had no effect on the proliferation of normal cells (L6 myoblasts and skin fibroblasts) at concentrations up to 100 μM [30].

Based on our results and published studies, further studies focused on pterostilbene at concentrations 10, 40, and 60 µM. Flow cytometric cell cycle analysis indicated that pterostilbene at all concentrations used reduced the G2/M population of HT-29 cells. Treatment with pterostilbene at lower concentrations induced an increase in the G0/G1 phase, indicating that it delayed the progression of the cell cycle in HT-29. The highest concentration of pterostilbene increased the cell population in sub-G1 cells. Therefore, these results indicate the dose-dependent ability of pterostilbene to induction of HT-29 cell cycle arrest and apoptotic cell death.

Cell cycle arrest is one of the prerequisites for cell differentiation. Poorly differentiated colon cancer cells are more aggressive. Therefore, differentiation therapy of cancer has been attracting considerable interest and is based on targeting uncontrolled growth and repairing the cancer cells’ differentiation and cell death programs [31]. Chakraborty et al. [32] showed that exposure to pterostilbene inhibits the growth of MCF-7 breast cancer due to differentiation of mammary carcinoma cells and activation of autophagy. A ubiquitous biochemical marker of colonocyte differentiation is the high activity of the enzyme alkaline phosphatase (ALP) [33]. While differentiated colon epithelial cells express relatively high levels of the enzyme, the activity of ALP in poorly differentiated colon cancer cells is low or negligible [34]. Some previous studies showed that colon cancer cell growth inhibition caused by natural polyphenols (e.g., resveratrol, ellagic acid, quercetin) was accompanied by increased activities of the differentiation markers alkaline phosphatase and dipeptidyl peptidase [35]. The current study revealed that pterostilbene at all concentrations used had no influence on ALP activity in HT-29 cells. Thus, we suggest that the mechanism of pterostilbene activity in HT-29 cells is not associated with stimulation of their differentiation.

To understand the molecular mechanisms underlying the growth inhibition induced by pterostilbene, the expression of genes encoding signaling proteins and the level of those proteins were performed. Although the primary target of pterostilbene is still unknown, studies revealed that it could influence multiple signaling pathways critical to cancer development. The pathogenesis of colorectal cancer is complex and heterogeneous, with the involvement of multiple cellular transduction cascades, including AKT and STAT3 pathways. Li et al. [36] found out that resveratrol inhibited colon cancer cell proliferation, induced G1 phase arrest, and cell apoptosis by inhibiting AKT kinase and its downstream signaling targets. Additionally, it has been shown that AKT served as an upstream regulator of STAT3. In our study, we aimed at evaluating whether pterostilbene, a natural analog of resveratrol, could exert its anticancer activity against human colon cancer cells through the alteration of the AKT and STAT3 signaling pathways. 

AKT kinase is a key intermediate of signaling that promotes colon cancer cell progression, prevents apoptosis, and increases cell proliferation [37]. Activation of the AKT kinase regulates cyclin stability and inhibits cyclin-dependent kinase inhibitors such CDKN1A (p21^Waf1/Cip1^) and CDNK1B (p27^Kip1^) [38]. To elucidate the mechanism of pterostilbene antiproliferative activity on HT-29 cells, we investigated whether pterostilbene modulates cell cycle progression by regulation of AKT kinase activity and expression of genes encoding cyclin D1 and p21. Our results showed a dose-dependent inhibitory effect of pterostilbene on the expression and activity of AKT kinase. Moreover, pterostilbene at concentrations 40 and 60 µM significantly decreases the transcriptional activity of the gene encoding cyclin D1, suggesting the disruption of the uncontrolled progression of the cell cycle of these cells. Furthermore, pterostilbene at concentrations ≥40 µM increased the expression of the *CDKN1A* gene that encodes p21 protein. The changes in *CDKN1A* mRNA were accompanied by an up-regulation of p21 expression in HT-29 cells incubated with pterostilbene at concentrations 40 and 60 µM. Based on the literature data, up-regulation of p21 expression blocks the transition of cells from the G1 to S phase [39]. In this context, it can be suggested that one of the possible mechanisms of the biological activity of pterostilbene against HT-29 cells is related to the inhibition of proliferation through the down-regulation of the AKT signaling pathway. 

The cellular mechanisms contributing to colorectal cancer are still not well understood but involve signaling protein dysregulation, which also includes STAT3 activation. The signal transducer and activator of transcription 3 (STAT3) is one of the main oncogenic pathways and is involved in the control of transcription of several cell cycle and proliferation-associated genes [40]. Activation of STAT3 is frequently detected in primary human colorectal carcinoma cells, as well as established human colorectal cancer cell lines, and is commonly associated with a worse prognosis [41,42,43]. Elevated levels of STAT3 phosphorylation were correlated with tumor invasion, metastasis, and the stage [44]. Many studies revealed the association between STAT3 activation, up-regulated expression of Cyclin D1, c-Myc, and survivin, and cell cycle progression in colon cancers [45,46]. Inhibition of the STAT3 signaling pathway in cancer cells has been shown to result in inhibition of growth and induction of apoptosis, making it an attractive therapeutic target for colorectal carcinoma [44]. There is increasing evidence that some phytochemicals could be potential candidates for the treatment and prevention of colorectal cancer, in part by suppressing the activation of the STAT3 pathway [47,48]. 

To explore the mechanism of the biological activity of pterostilbene on HT-29 colon cancer cells, we also examined its effect on the expression and activity of STAT3. Surprisingly, the expression of the *STAT3* gene increased in HT-29 cells treated with the stilbene for 6 h; however, at 12 h, no significant changes in transcriptional activity of the *STAT3* gene were observed, so the effect on *STAT3* transcription was temporary. Notwithstanding the slight increase of STAT3 expression at 3 h, pterostilbene at all studied concentrations markedly downregulated STAT3 activation in HT-29 cells compared with control. Thus, the results of our studies suggest that pterostilbene suppresses proliferation and induces apoptosis of HT-29 colon cancer cells through down-regulation of both AKT and STAT3 pathways. A similar mechanism of pterostilbene activity was observed in human pancreatic BxPC-3 and MIA PaCa-2 cell lines [49]. 

As observed, pterostilbene demonstrated a potent growth inhibitory effect. In our study, we detected a decrease in DNA synthesis and cell cycle arrest; the high reduction in cell number after pterostilbene treatment could only be understood if cell death mechanisms are activated. Pterostilbene may exhibit cancer cell death through a different mechanism of cell death (e.g., apoptosis and autophagy), but apoptosis has been postulated as the main cell death program activated by pterostilbene [50,51,52]. In order to determine whether observed cell death was apoptotic, the possible impact of pterostilbene on *BAX* gene expression and caspase 3 activity was evaluated. The findings of this study revealed that pterostilbene significantly enhanced the transcriptional activity of the *BAX* gene in HT-29 colon cancer cells at all concentrations used. Furthermore, pterostilbene at a concentration of 60 µM increases the activity of caspase 3. Activated caspase 3 is considered as the most important of executioner caspases and plays the main role in the breakdown of the nucleus and other cellular compartments during apoptosis [53]. The enhanced caspase 3 activity is correlated with the increase in the number of dead (sub-G1) cells determined by flow cytometry. Moreover, pterostilbene at concentrations 40 and 60 µM induced an increase in the level of oligonucleosomal DNA fragmentation. Based on the increase in the sub-G1 phase and the enrichment of nucleosomes in the cytoplasm, we believe that pterostilbene induces apoptosis in HT-29 cells, which is consistent with other reports showing that pterostilbene induces G0/G1 cell cycle arrest in HL-60 leukemia cells [54] and stimulates nuclear condensation in MCF-7 and Bcap-37 breast cancer cells [55]. It also reported that pterostilbene induced externalization of phosphatidylserine in breast cancer cells [55]. The capability of pterostilbene to effectively inhibit the cancer cell viability through cell cycle arrest and apoptosis was also confirmed in lung cancer cells [56]. 

Recently, pterostilbene has been shown to induce both apoptosis and autophagy in bladder [51], lung [56], and breast [55] cancer cells. Autophagy plays a dual role in cancerogenesis. It is a major intracellular degradation mechanism used for responding to stress conditions to promote survival during starvation or lead to programmed cell death type II [56,57]. While originally autophagy was identified as a cell survival mechanism, recent studies revealed that it can act as a tumor suppressor because it plays context-specific roles in mediating cancer cell death [58]. The role of pterostilbene as a modulator of autophagy was observed by Siedlecka-Kroplewska et al. [54], which demonstrated pterostilbene-induced autophagic vacuoles accumulation in leukemia cells followed by cell death. Studies performed on lung cancer cells indicated that pterostilbene induced apoptosis after 48 h of incubation, whereas autophagy was observed in cells incubated with it for 24 h together with an increase in LC3-II expression [56]. Results published by Ko et al. [59] demonstrate a similar effect of pterostilbene on oral cancer cells. The authors not only observed that the cytotoxic effect of pterostilbene was mediated by cell cycle arrest and apoptosis but also demonstrated the ability of pterostilbene to induce autophagy confirmed by the formation of acidic vesicular organelles and the expression of LC3-II. Furthermore, they elucidated that autophagy-induced death of oral cancer cells incubated with pterostilbene was triggered by inhibition of AKT, ERK1/2, and p38. In our study, we also evaluated whether pterostilbene could affect the expression of genes related to autophagy. We estimated the transcriptional activity of genes that encode proteins that are essential for different steps of autophagy. The ULK1 protein is required for the initialization of autophagy, AMBRA1 is important for the activation of Beclin 1 and is an important factor at the crosstalk between autophagy and apoptosis, activation of Beclin1 plays an important role in a critical step of the autophagic process, autophagosome formation, by interacting with the class III-type phosphoinositide 3-kinase while LC3A is a structural protein of the autophagosome membrane [60,61,62]. Marked increases in transcriptional activities of *ULK1* (at 6 h), *AMBRA1* (at 12 h), and *LC3A* (at 12 h) mRNAs were observed in HT-29 cells treated with pterostilbene at concentration 60 µM. Surprisingly, pterostilbene did not affect gene encoding beclin 1 (*BCLN1*) expression at both studied time periods. The transient increase in *ULK1* mRNA at 6 h could be the consequence of autophagy induction. Published studies revealed that ULK1 levels are finely regulated during autophagy at transcriptional, translational, and degradation levels. The amount of ULK1 protein decreases during the first few hours of autophagy. The rescue of ULK1 requires constant transcription of the *ULK1* mRNA gene and de novo protein synthesis reactivation. Therefore, while the ULK1 protein is degraded, the *ULK1* mRNA is actively transcribed [63,64,65]. At a longer time period (12 h), we observed the increase in transcriptional activity of the *AMBRA1* and *LC3A* genes, suggesting that subsequent stages of autophagy were occurring in HT-29 cells. These findings suggest the ability of pterostilbene induction of colon cancer cells autophagy, but more detailed studies are needed to clarify whether that possible autophagy occurred as an early stage of pterostilbene-induced apoptosis in colon cancer cells.

In summary, the findings suggest that pterostilbene can effectively inhibit the growth and induce the death of human HT-29 colon cancer cells. The results presented suggest that the mechanism of the biological activity of pterostilbene is related to the downregulation of the STAT3 and AKT kinase pathways in HT-29 cells. These findings suggest that pterostilbene might be a promising cancer prevention and/or therapeutic agent. Further research and clinical trials are warranted to fully elucidate the effects of pterostilbene on human cancer.

## 4. Materials and Methods

### 4.1. Cell Line and Culture Conditions

The HT-29 human colon adenocarcinoma cell line was purchased from the American Type Culture Collection (ATCC, Rockvile, MD, USA). Cells were routinely grown in MEM medium (Sigma-Aldrich, St. Louis, MO, USA), supplemented with 10% fetal bovine serum (FBS; BioWest, Nualillé, France), 100 U/mL penicillin, 100 µg/mL streptomycin (Sigma-Aldrich), and 10 mM HEPES (Sigma-Aldrich). Cell cultures were as a monolayer at 37 °C in a humidified atmosphere containing 5% CO_2_. Cells were treated with pterostilbene solutions and proceeded as described below.

### 4.2. Preparation of Pterostilbene Stock Solution

Pterostilbene was purchased from Sigma-Aldrich. The stock solution of pterostilbene was prepared in dimethyl sulfoxide (DMSO) (Sigma-Aldrich) and further diluted in a sterile culture medium to the desired concentrations immediately before use. The final DMSO concentration in the working solutions was 0.1%.

### 4.3. Cell Growth Determination

The effect of pterostilbene on HT-29 cell growth was analyzed by In Vitro Toxicology Assay Kit, Sulforhodamine B (SRB) based (Sigma-Aldrich). Cells were seeded at an initial density of 2 × 10^3^ cells/well in 200 µL MEM medium supplemented with the components given above and allowed to attach and grow. After 24 h, the medium was aspirated, and cells were exposed to freshly prepared medium containing pterostilbene (5, 10, 20, 40, 50, 60, 75, and 100 µM) for 48 h. At the end of the incubation period, cells were fixed in 10% trichloroacetic acid (4 °C, 1 h) and stained with SRB. After the liberation of the incorporated dye by the addition of 10 mM Tris-HCl, the absorbance was measured at 570 nm and 690 nm (reference wavelength) using the Labtech LT-5000c multiplate reader. The growth of treated cells was expressed as a percentage of untreated control cells. 

### 4.4. Cell Proliferation Assay 

The effect of pterostilbene on HT-29 cell proliferation was evaluated by determining BrdU incorporation using the 5-bromo-2-deoxyuridine (BrdU) colorimetric enzyme-linked immunosorbent assay (ELISA) kit (Roche, Mannheim, Germany). HT-29 cells were seeded in 96-well plates at density 8 × 10^3^ cells/well in 200 µL medium followed by overnight incubation. Afterward, the media were replaced with fresh ones containing pterostilbene (5, 10, 20, 40, 50, 60, 75, and 100 µM). The BrdU labeling solution was added to the medium for the last 4 h of incubation. The incorporation rate of BrdU was determined after 24 and 48 h. After the labeling medium was removed, cells were fixed, and DNA was denatured with FixDenat solution. Immune complexes were formed using peroxidase-conjugated antibodies. The incorporation of BrdU was determined by measuring the absorbance at λ = 450 nm (with reference λ = 690 nm) using a microplate spectrophotometer Labtech LT-5000c (Labtech International Ltd., Uckfield, UK).

### 4.5. Alkaline Phosphatase Activity Assay

As a measure of cell differentiation, alkaline phosphatase (ALP) activity was determined in cell lysates by a commercially available kit (BioMaxima, Lublin, Poland). HT-29 cells were plated on 56.7 cm^2^ dishes (1.1 × 10^6^ cells/dish) and allowed to grow for 2 days before being exposed to medium supplemented with pterostilbene (10, 40, and 60 µM) or sodium butyrate (NaB 1mM) for 3 days. Subsequently, cells were harvested in cold PBS solution (pH 7.4) followed by one freeze-thawed cycle and sonication. The ALP activity was determined spectrophotometrically (λ = 405 nm) in cell lysates using para-nitrophenyl phosphate as substrate and calculated from a para-nitrophenol standard curve. The results were expressed relative to cellular protein content, which was determined spectrophotometrically by the Bradford assay with bovine albumin as a standard.

### 4.6. Cell Cycle Analysis

To study the cell cycle distribution, HT-29 cells were seeded at a density of 1 × 10^5^ cells onto 21.5 cm^2^ and allowed to attach and grow for 24 h. Cell cultures were then exposed to various concentrations (0, 10, 40, and 60 µM) of pterostilbene. After 48 h, cells were trypsinized, washed twice in ice-cold PBS buffer, and fixed in 70% ice-cold ethanol while vortexing at low speed. The samples were stored at −20 °C overnight. Directly before the assay, cells were incubated with RNase A (final concentration, 200 µg/mL) in PBS buffer for 1 h at 37 °C in the dark and then stained with propidium iodide solution (Sigma-Aldrich, final concentration, 10 µg/mL). The DNA content and cell cycle distribution of the cells were analyzed by BD FACS Aria II flow cytometer and BD FACSDiva software (BD Biosciences, San Jose, CA, USA). 

### 4.7. Total RNA Extraction and Quantitative Real-Time RT-PCR (RT-qPCR)

To determine the effect of pterostilbene on the transcriptional activity of genes encoding proteins associated with cell cycle regulation (*CCND1*; *CDKN1A*), signaling pathways (*AKT*; *STAT3*), apoptosis, and autophagy (*ULK1*; *AMBR1*; *BCLN1*; *LC3A*), cells were seeded at a density of 8 × 10^5^ onto 21.5 cm^2^ culture dishes. One day after plating, the media were changed, and cells were incubated with pterostilbene (10, 40, and 60 µM) for 6 and 12 h. The pterostilbene untreated cells were used as a control. Total RNA was extracted from cells using TRI REAGENT (Zymo Research, Irvine, CA, USA) according to the manufacturer’s specifications. RNA concentration and purity were checked using the Shimadzu UV-1800 spectrophotometer (Shimadzu, Kyoto, Japan). Samples showing a ratio of Abs 260/280 nm between 1.8 and 2.0 were only used for experiments. Detection of the expression of examined genes was carried out using an RT-qPCR technique with an SYBR Green chemistry (SYBR Green Quantitect RT-PCR Kit) (Qiagen Inc., Valencia, CA, USA) and CFX Connect Real-Time PCR Detection System (Bio-Rad, Hercules, CA, USA). Aliquots (0.1 μg) of total cellular RNA were applied to one-step RT-qPCR in a 20 μL reaction volume. The primers for all genes studied were commercially available (Sigma-Aldrich). The thermal profile for RT-qPCR was as follows: 50 °C for 30 min for reverse transcription and 95 °C for 15 min, followed by 45 cycles at 94 °C for 15 s, 55 °C for 30 s, and 72 °C for 45 s for amplification. Each gene analysis was performed in triplicate. The mRNA copy numbers of the examined genes were determined on the basis of the commercially available standard of β-actin (TaqMan DNA Template Reagent Kit, Invitrogen, Waltham, MA, USA) and recalculated per μg of total RNA. The transcriptional activity of genes in cultured cells was expressed as the fold change relative to the corresponding controls. The value of fold change > 1 reflects increased expression of the target gene, and a value of fold change < 1 points to a decrease in the gene expression.

### 4.8. Measurement of the p21 Protein Level

To determine the concentration of p21 protein, HT-29 cells were seeded at a density of 5.5 × 10^6^ per 100-mm diameter dish and cultured for 24 h. Afterward, cells were exposed to pterostilbene at concentrations of 10, 20, and 60 µM for 24 h. Subsequently, cells were washed with ice-cold PBS, scraped from the dishes, and centrifuged. They were lysed on ice in cell extraction buffer. The expression of p21 was determined with a commercially available ELISA kit (Invitrogen) following the manufacturer’s instruction. Absorbance was measured using the Labtech multiplate reader at λ = 450 nm. p21 concentrations were normalized to the total protein content in cells, as measured by the Bradford assay.

### 4.9. The AKT Activity Assay 

The level of total and phosphorylated AKT proteins in HT-29 cells were determined simultaneously with the ELISA kit (RayBio, Norcross, GA, USA). HT-29 cells were seeded (7 × 10^5^ cells) and left to adhere. Subsequently, cells were incubated with pterostilbene at concentrations of 10, 40, and 60 µM for 24 h. The cells were then lysed, and the contents of both AKT and p-AKT were measured according to the manufacturer’s protocol. Absorbance at 450 nm was measured. The results obtained were calculated as the ratio of p-AKT/total AKT and expressed as a percentage of the control.

### 4.10. The STAT3 Phosphorylation Assay

To verify the effect of pterostilbene on STAT3 activation, the level of total and phosphorylated STAT3 proteins was determined simultaneously with a commercially available ELISA kit (Abcam, Cambridge, UK). HT-29 cells were seeded at a density of 1.5 × 10^5^ cells/mL and left to adhere. Afterward, cells were incubated with pterostilbene at concentrations of 10, 40, and 60 µM for 24 h. Then, the cells were lysed, and the contents of both total STAT3 and phospho-STAT3 (p-STAT3) were measured according to the vendor’s protocol. The results obtained were calculated as a ratio of p-STAT3/total STAT3 and expressed as a percentage of the control.

### 4.11. Caspase Activity Assay

To determine the proapoptotic caspase 3 activity, the “Caspase-3 Assay Kit, Colorimetric” (Sigma-Aldrich) was used. Cells were plated at a density of 1 × 10^6^ per 100-mm diameter dish and cultured for 24 h. Subsequently, the cells were treated with pterostilbene (10, 40, and 60 µM) for 48 h. After this period, cells were scraped from the dishes, lysed, centrifuged, and stored at −80 °C. The assay was based on the hydrolysis of the substrate, Ac-DEVD-pNA (acetyl-Asp-Glu-Val-Asp-pNA), by active caspase 3. The absorbance of released p-nitroaniline (pNA) was measured at 405 nm using the multiplate reader Labtech LT-5000c. The results were expressed relative to cellular protein content, which was determined spectrophotometrically using the Bradford assay.

### 4.12. DNA Fragmentation Assay

The Cell Death Detection ELISA kit (Roche) was used to detect the occurrence of nuclear DNA fragmentation. Colon cancer cells were seeded into 96-well tissue culture plates (1 × 10^4^ cells/well in 200 µL cell culture medium) and allowed to attach and grow. After 24 h, the medium was aspirated, and cells were exposed to the freshly prepared medium containing pterostilbene (10, 40, and 60 µM) for 48 or 72 h. According to the manufacturer’s instructions, adherent cells were lysed and centrifuged to produce a nucleosome-containing supernatant. The enrichment of mono- and oligonucleosomes released into the cytoplasm of cell lysates was detected by biotinylated anti-histone- and peroxidase-coupled anti-DNA-Ab and was calculated by using the formula: absorbance of sample cells/absorbance of control cells. The enrichment factor was used as a parameter of apoptosis. Colorimetric results were measured using a multiplate reader at a wavelength of 405 nm.

### 4.13. Statistical Analysis

Statistical analysis was performed with the use of Statistica PL, ver. 12.0 Software (StatSoft Polska, Cracow, Poland). The experiments were repeated three times with at least triplicated samples. All results were expressed as means ± standard deviation (SD). Data were analyzed by the Student’s *t*-test or by one-way analysis of variance (ANOVA) followed by a post hoc Tukey’s test to evaluate significance between examined groups. Differences with a probability (*p*) value less than 0.05 were considered statistically significant.

## Data Availability

Not applicable.

## Sample Availability

Samples of the compounds are not available from the authors.
